# Availability of eleven species names of *Eupelmus* (Hymenoptera, Eupelmidae) proposed in Al khatib et al. (2014)

**DOI:** 10.3897/zookeys.505.9021

**Published:** 2015-05-25

**Authors:** Fadel Al Khatib, Lucian Fusu, Astrid Cruaud, Gary Gibson, Nicolas Borowiec, Jean-Yves Rasplus, Nicolas Ris, Gérard Delvare

**Affiliations:** 1 INRA, Univ. Nice Sophia Antipolis, CNRS, UMR 1355-7254 Institut Sophia Agrobiotech, 06900 Sophia Antipolis, France; 2 INRA, UMR 1062 CBGP, 755 avenue du Campus Agropolis, Montferrier-sur-Lez Cedex, France; 3 CIRAD, UMR 55 CBGP, 755 avenue du campus Agropolis, Montferrier-sur-Lez, Cedex, France; 4 Faculty of Biology, Alexandru Ioan Cuza University, Iasi, Romania; 5 Agriculture and Agri-Food Canada, Canadian National Collection of Insects, Arachnids and Nematodes, Ottawa, Canada

**Keywords:** Eupelmus, ICZN, systematics, taxonomy

## Abstract

This paper is an addendum for the availability of the names of 11 new species proposed in Al khatib et al. (2014).

## Introduction

Al khatib et al. (2014) described 11 new species of West Palaearctic *Eupelmus*, but only the acronyms of the depositories where the holotypes are deposited were given in the publication. The full names and location of the depositories were provided, but only in a Word document (Appendix S2) as part of the Supplementary Information (SI) available on the website of *Systematic Entomology* (http://onlinelibrary.wiley.com/doi/10.1111/syen.12089/suppinfo). Article 16.4 of the International Code of Zoological Nomenclature states that “Every new specific and subspecific name published after 1999,... must be accompanied in the original publication... where the holotype or syntypes are extant specimens, by a statement of intent that they will be (or are) deposited in a collection and a statement indicating the name and location of that collection” (ICZN 2012). A museum acronym is not a statement of the location or even necessarily a clear indication of the name of a collection. Further, Appendix S2 cannot be considered as part of the publication or itself a publication because it is a Word document and thus contravenes ICZN Article 8.1.3, in addition to Articles 8.5.2 and 8.5.3. As a result, the new names published in Al khatib et al. (2014) are not available because the complete name and locality information of the holotype depositories are not provided in the publication, but only in a document that supplements the publication. For this reason, the authors provide in the present paper the new names published in Al khatib et al. (2014) with a precise statement (Table 1) as to the name and location of the collection in which the holotypes and other material listed in other supplementary files are deposited. Accordingly, the availability of the 11 names proposed in Al khatib et al. (2014) and given below takes the date of the present publication, as per ICZN Article 10.1. Al khatib et al. (2014) also did not include in the publication paratype information for new species with numerous paratypes, citing this information only in Appendix S1 on the *Systematic Entomology* website. We therefore also include here paratype designations to ensure this information is validly published.

**Table 1.** Collection acronyms, names and locations for specimen deposition cited in Al khatib et al. (2014).

**AICF**	Lucian Fusu collection, A1. I. Cuza University, Iasi, Romania.
**BMNH**	The Natural History Museum, London, United Kingdom.
**CBGP**	Center for Biology and Management of Populations, Montpellier, France.
**CNC**	Canadian National Collection of Insects, Arachnids and Nematodes, Agriculture & Agri-food Canada, Ottawa, ON, Canada.
**FALPC**	Fadel Al khatib personal collection, Faculty of Agricultural Engineering, University of Aleppo, Syria.
**GDPC**	Gérard Delvare personal collection, Montpellier, France.
**MKUI**	Plant Protection Department, Mustafa Kemal University, Antakya-Hatay, Turkey.
**MNHG**	Museum of Natural History of Geneva, Switzerland.
**MNHN**	National Museum of Natural History, Paris, France.
**NHMW**	Naturhistorisches Museum, Wien, Austria.
**NHRS**	Naturhistoriska riksmuseet, Stockholm, Sweden.
**NMPC**	Narodni Muzeum v Praze, Prague, Czech Republic.
**RMNH**	Rijksmuseum van Naturrlijke Historie collection, Naturalis Biodiversity Centre, Leiden, The Netherlands.

## Results

### Eupelmus (Eupelmus) confusus

Taxon classificationAnimaliaHymenopteraEupelmidae

Al khatib

http://zoobank.org/E9A1F8A3-00D1-4DB6-9A04-5ACBEBDBFEA3

Eupelmus (Eupelmus) confusus Al khatib et al. (2014): 822–828.

#### Type material.

**Holotype** ♀. **FRANCE:** Var, Fayence, 43.61774°N, 06.69774°E, 17.iii.2012, emerged 25.iii.2012, ex *Diplolepis rosae* on *Rosa canina* (N. Ris) (1 ♀) [FAL1195/10206] (in MNHG). **Paratypes. CYPRUS: Lemesos**, 6 km N of Lemesos, 24–25.V.2009, N34.73189°, E33.05175°, pods of carob tree with *Apomyelois ceratoniae* & *Asphondylia gennadii* (Fusu L. & Popovici O.) (5 ♀ 6 ♂ not sequenced) (in AICF) (1 ♀) [LF.ma.CY 01/10427] (in AICF); **6 km N of Lemesos**, 25. V. 2009, sweep net, N34.727028°, E33.052278° (Fusu L. & Popovici O.) (1 ♀ 10 ♂ not sequenced)(in AICF). **FRANCE: Alpes-Maritimes**, Biot, N43.63455°, E7.08249°, 11.iii.2012, emerged 27.iii.2012, ex *Andricus kollari* on *Quercus pubescens* (N. Ris) (2 ♀) [FAL1227/10215, FAL1227/10216] (in FALPC); **Alpes-Maritimes**, Opio, N43.64479°, E6.99957°, 04.x.2012, (F. Al khatib & P. Gory) (1 ♀) [FAL1485/10313] (in FALPC); **Alpes-Maritimes**, Pégomas, N43.58844°, E6.93612°, 08.vi.2012, emerged 11.vi.2012, ex *Myopites stylata* on *Dittrichia viscosa* (F. Al khatib & P. Gory) (1 ♀) [FAL1429/10433] (in GDPC); **Alpes-Maritimes**, Sophia-Antipolis, N43.62443°, E7.03667°, 21.ii.2012, emerged 27.ii.2012, ex *Myopites stylata* on *Dittrichia viscosa* (F. Al khatib) (1 ♀ 1 ♂) [FAL1029/10142, FAL1032/10432] (in MNHG); **Alpes-Maritimes**, Sophia-Antipolis, N43.61669°, E7.03722°, 07.vi.2012, emerged 16.vi.2012, ex *Biorhiza pallida* on *Quercus pubescens* (F. Al khatib & P. Gory) (1 ♀) [FAL1338/10227] (in MNHN); **Alpes-Maritimes**, Villars-sur-Var, N43.93730°, E7.08068°, 14.iii.2012, emerged 18.iii.2012, ex *Diplolepis rosae* on *Rosa canina* (F. Al khatib & N. Ris) (2 ♀) [FAL1198/10209 (in MNHG), FAL1198/10210 (in FALPC)]; **Ardèche**, Saint-Georges-les-Bains, N44.85028°, E4.82433°, 13.vi.2012, emerged 14.vi.2012, ex *Biorhiza pallida* on *Quercus pubescens* (F. Al khatib & M. Thaon) (2 ♀) [FAL1325/10224, FAL1325/10225] (in CBGP); **Ardèche**, Saint-Georges-les-Bains, N44.85028°, E4.82433°, 13.vi.2012, emerged 11.vii.2012, ex *Dryocosmus kuriphilus* on *Castanea sativa* (M. Thaon) (1 ♀ 1 ♂) [NB489/10418, NB489c/10419] (in GDPC); **Ardèche**, Saint-Georges-Montpellier, N43.6104°, E3.77227°, ix.2011, emerged ix.2011, ex *Bactrocera oleae* on *Olea europaea* (L. Brancaccio & M. Thaon) (1 ♀ 1 ♂) [FAL1278/10443, FAL1280/10445] (in MNHN); **Aude**, Durban-Corbières, N42.99825°, E2.80690°, 27.iii.2012, emerged 31.iii.2012, ex *Myopites stylata* on *Dittrichia viscosa* (F. Al khatib & N. Ris) (1 ♀) [FAL1122/10175] (GDPC); **Bouches-du-Rhône**, La Ciotat, garden, 09.I.2011 emerged 13-30.IV.2011, *Lasioptera carophila* on *Foeniculum vulgare* (H. Dumas) (5 ♀ 6 ♂ not sequenced, in AICF) (1♀) [LF.ma.FR 01/10422] (in AICF); **Gard**, Garons, N43.76371°, E4.42588°, 11.i.2012, emerged 27.ii.2012, ex *Myopites stylata* on *Dittrichia viscosa* (N. Ris) (1 ♀) [FAL1092/10162] (in MNHG); **Gard**, Roquemaure, N44.03148°, E4.72747°, x.2011, emerged x.2011, ex *Bactrocera oleae* on *Olea europaea* (N. Borowiec) (1 ♀) [FAL1274/10447] (in CBGP); **Haute-Corse**, Aléria, N42.12861°, E9.46555°, 22.ix.2011, ex seeds of *Asphodelus ramosus* infested by *Bruchophagus* sp. (J. Balajas) (2 ♀ 1 ♂) [GDEL4111/10187, GDEL4111/10188, GDEL4111/10189] (in MNHG); **Haute-Corse**, Lumio, N42.55879°, E8.81299°, 23.ix.2012, emerged 28.ix.2012, ex *Bactrocera oleae* on *Olea europaea* (F. Ceccaldi) (2 ♀) [FAL1519/10411, FAL1519/10412] (MNHN); **Haute-Corse**, Piedicorte di Gaggio, N42.22166°, E9.26527°, 22. ix.2011, ex seeds of *Asphodelus ramosus* infested by *Bruchophagus* sp. (J. Balajas) (2 ♀ 3 ♂) [GDEL4114/10190, GDEL4114/10191, GDEL4114a, GDEL4114b & GDEL4114c] (in MNHN); **Hérault**, Causses-et-Veyran, N43.47131°, E3.08508°, x.2011, emerged x.2011, ex *Bactrocera oleae* on *Olea europaea* (A. Auguste-Maros) (1 ♀) [FAL1254/10453] (in FALPC); **Hérault**, Frontignan, N43.43926°, E3.74145°, 17.vi.2012, emerged 19.vi.2012, ex *Myopites stylata* on *Dittrichia viscosa* (F. Al khatib & N. Ris) (1 ♀) [FAL1446/10309] (FALPC); **Hérault**, Laroque, 250-400 m, N45.91722°, E3.74361°, 05.vii.2013, sweeping on *Quercus pubescens* (G. Delvare), (1 ♀) [4173/10596] (in GDPC); **Hérault**, Mèze, N43.41670°, E3.6000°, x.2011, emerged x.2011, ex *Bactrocera oleae* on *Olea europaea* (N. Ris) (2 ♀) [FAL1257/10454 (in CNC), NB229/7052 (in FALPC)]; **Monaco**, Monaco, N43.73263°, E7.41369°, x.2010, ex *Bactrocera oleae* on *Olea europaea* (J.-C. Malausa & C. Roques) (1 ♀) [FAL1247/10436] (in CNC); **Pyrénées-Orientales**, Argelès-sur-Mer, N42.581000°, E3.010910°, x.2011, emerged x.2011, ex *Bactrocera oleae* on *Olea europaea* (N. Ris) (4 ♀ 1 ♂) [FAL1255/10449 & FAL1255/10450 (in GDPC), NB362v/7078, NB362w/7079, FAL1256/10451 (in FALPC)]; **Pyrénées-Orientales**, Banyuls-sur-Mer, 04.ii.2012, emerged 20.ii.2012, ex *Myopites stylata* on *Dittrichia viscosa* (J. Lecomte) (1 ♀) [FAL1100/10164] (in CBGP); **Pyrénées-Orientales**, Banyuls-sur-Mer, N42.47194°, E3.14333°, 250 m, 21.iii.2010 ex galls of *Timaspis phoenixopodos* on *Lactuca viminea* (G. Delvare & J. Lecomte) (2 ♀) [GDEL4001/3303, GDEL4002/3296] (in GDPC); **Pyrénées-Orientales**, Banyuls-sur-Mer, N42.46972°, E3.12388°, 10 m, 21.iii.2010 ex galls of *Timaspis phoenixopodos* on *Lactuca viminea* (G. Delvare & J. Lecomte) (1 ♀) [GDEL4003/3302] (in GDPC); **Pyrénées-Orientales**, Calce, N42.7348°, E2.75471°, x.2011, emerged x.2011, ex *Bactrocera oleae* on *Olea europaea* (N. Borowiec & L. Brancaccio ) (2 ♂) [FAL1251/10448 (in GDPC), FAL1251/10283 (in FALPC)]; **Pyrénées-Orientales**, Perpignan, N42.67720°, E2.86912°, 18.vi.2012, emerged 19.vi.2012, ex *Myopites stylata* on *Dittrichia viscosa* (F. Al khatib & N. Ris) (1 ♀) [FAL1455/10312] (in FALPC); **Var**, Rians, N43.57352°, E5.77148°, 31.ii.2012, emerged 09.iii.2012, ex *Diplolepis rosae* on *Rosa canina*, (N. Ris) (2 ♀ 1 ♂) [FAL1204/10212 (in CNC), FAL1204/10213 & FAL1205/10280 (in MNHG)]; (1 ♀) [FAL1195/10207] (in MNHN), same data as holotype. **GREECE: Seres**, Kerkini Lake Nat.Park, Kerkini Mts near Vironeia, 300 m, N41.27833°, E23.21955°, sweep net 22.VI.2008 (Fusu, Popovici & Ramel) (1 ♀) (in AICF) (1 ♀) [LF.ma.GR 01/10425] (in AICF); **Seres**, Kerkini lake, Krousia Mts. Site, N41.20180°, E23.07747°, Malaise trap, 12-18.IX.2007 (G. Ramel) (1 ♀) [LF.ma.GR 02/10426] (in AICF); **Seres**, Kerkini Mts., Plateaux Beech, N41.28580°, E23.03368°, Malaise trap, 08.VIII to 13.VIII.2007, (G. Ramel) (1 ♀ not sequenced, in AICF); **Seres**, Kerkini Lake N. Park, nr Kerkini, Pumping St. Site, N41.19760°, E23.08883°, 13.VI to 19.VI.2007, Malaise trap (G. Ramel) (1♀ not sequenced, in AICF); **Seres**, Kerkini Lake N. Park, Kerkini, Krousia Mts site, 190 m, N41.20180°, E23.07747°, 06.VI-12.VI.2007, Malaise tr. (G. Ramel) (4 ♀ not sequenced, in AICF); same data but 13.VI-19.VI.2007 (2 ♀ not sequenced, in AICF); same data but 20.VI-26.VI.2007 (4 ♀ not sequenced, in AICF). **IRAN: Kerman Prov.**, Bidkhan, 2897 m, N29°34.956’ E 56°30.612’, 11.v.2012, ex galls on *Salix alba* (M. Mahdavi) (1 ♀) [LF.ma.IR 05/10424] (in AICF). **ITALY: Liguria**, Bussana-Vecchia, N43.84026°, E7.82905°, 02.i.2012, emerged 20.ii.2012, ex *Myopites stylata* on *Dittrichia viscosa* (E. Spagnol) (4 ♀) [FAL1051/10145 (in GDPC), FAL1088/10154 & FAL1063/10149 (in CBGP), FAL1074/10153 (in MNHN)] and (3 ♂ not sequenced) [FAL1077a, FAL1077b, FAL1077c] (in MNHN); (3 ♀ not sequenced) [FAL1422d & FAL1422c, FAL1422b] (in FALPC) and (2 ♂ not sequenced) [FAL1418a, FAL1418b] (in CNC) same data but 06.vi.2012, emerged 07.vi.2012 (N. Ris). **SPAIN: Logroño**, La Rioja, 15.iii.2012, emerged 16.iii.2012, ex *Myopites stylata* on *Dittrichia viscosa* (R. Cantera Rioja) (2 ♀) [FAL1108/10250 (in GDPC), FAL1110/10168 (in FALPC)]. **SWEDEN: Skåne**, Sk, Höganäs kommun, Kullabergs naturreservat, between Hjortstugan and Ransvik, Oak forest in southern slope, N56.29421°, E12.48399°, 27.vi to 30.vii.2005, Trap ID 1004, Coll. event 1797 (SMTP) [LF.u.SW.03/10660] (in NHRS).

### Eupelmus (Eupelmus) gemellus

Taxon classificationAnimaliaHymenopteraEupelmidae

Al khatib

http://zoobank.org/4BCB5C66-7AC7-431B-8F51-C76CF7D56AD4

Eupelmus (Eupelmus) gemellus Al khatib et al. (2014): 828–837.

#### Type material.

**Holotype** ♀. **FRANCE:** Haute-Corse, Calenzana, 11.ix.2012, emerged 18.ix.2012, ex *Bactrocera oleae* on *Olea europaea* (F. Ceccaldi) (1 ♀) [FAL1515/10408] (in MNHG). **Paratypes. FRANCE: Alpes-Maritimes**, Biot, N43.63455°, E7.082490°, 29.x.2012, emerged 01.iii.2013, ex *Megastigmus pistaciae* on *Pistacia lentiscus* (F. Al khatib & N. Ris) (2 ♀ 1 ♂) [FAL1522/10483, FAL1522/10484, FAL1522/10485] (in MNHN); **Alpes-Maritimes**, Biot, N43.63455°, E7.082490°, 11.iii.2012, emerged 27.iii.2012, ex *Mesophleps oxycedrella* on *Juniperus oxycedrus* (N. Ris), (2 ♀) [FAL1359/10230 (in MNHG), FAL1359/10231 (in AICF)]; **Alpes-Maritimes**, Mont-Chauve, 476 m N 43.76578°, E7.27024°, 01.xi.2012, emerged 18.xii.2012, ex *Bactrocera oleae* on *Olea europaea* (M. Thaon) (1 ♀) [NB29/10413] (in FALPC); **Alpes-Maritimes**, Sophia-Antipolis, N43.624423°, E07.03667°, 21.ii.2012, emerged 27.ii.2012, ex *Myopites stylata* on *Dittrichia viscosa* (F. Al khatib) (1 ♀) [FAL1089/10143] (in FALPC); **Ardèche**, Saint-Georges-Montpellier, N43.6104°, E3.77227°, x.2011, ex *Bactrocera olea* on *Olea europaea* (L. Brancaccio & M. Thaon) (1 ♀) [FAL1279/10444] (in MNHG); **Aude**, Bize-Minervois, N43.32692°, E2.870750°, 27.iii.2012, emerged 11.iv.2012, ex *Mesophleps oxycedrella* on *Juniperus oxycedrus* (F. Al khatib & N. Ris), (1 ♀) [FAL1360/10233] (in MNHN); **Aude**, Durban-Corbières, N42.99825°, E2.80690°, 27.iii.2012, emerged 06.iv.2012, ex *Mesophleps oxycedrella* on *Juniperus oxycedrus* (F. Al khatib & N. Ris), (1 ♀) [FAL1362/10234] (in FALPC); **Aude**, Gruissan, N43.12105°, E3.09539°, x.2011, ex *Bactrocera oleae* on *Olea europaea* (N. Ris) (1 ♀) [FAL1266/10446] (in CBGP); **Bouches-du-Rhône**, Lançon-de-Provence, 33 m, N43.54818°, E5.16727°, x.2011, ex *Bactrocera oleae* on *Olea europaea* (A. Auguste-Maros) (1 ♂) [FAL1269/10439] (in MNHN); **Bouches-du-Rhône**, La Ciotat, 53 m, N43.19011°, E5.65905°, x.2010, ex *Bactrocera oleae* on *Olea europaea* (A. Auguste-Maros) (1 ♂) [FAL1243/10437] (in MNHG); **Haute-Corse**, Bisinchi, 593 m, N42.48983°, E9.32797°, 18.vi.2012, emerged 03.vii.2012, ex *Dryocosmus kuriphilus* on *Castanea sativa* (N. Borowiec & M. Thaon) (4 ♀) [NB441/10414 (in MNHG), NB441/10415 & NB441/10416 (in FLAPC), NB441/10417 (in MNHN)]; **Haute-Corse**, Lumio, N42.55879°, E8.81299°, 13.i.2012, emerged 27.ii.2012, ex *Myopites stylata* on *Dittrichia viscosa* (F. Ceccaldi) (1 ♀) [FAL1013/10137] (in AICF); **Haute-Corse**, Muratu, 750 m, N42.5559°, E9.29929°, emerged 23.i.2013, ex *Dryocosmus kuriphilus* on *Castanea sativa* (N. Borowiec & M. Thaon) (1 ♀) [2013CYN355/10664] (in FALCP); **Var**, la Garde-Freinet, 366 m, N43.31597°, E6.47534°, emerged 28.ii.2013, ex *Dryocosmus kuriphilus* on *Castanea sativa* (N. Borowiec & M. Thaon) (1 ♂) [2013CYN448/10663] (in FALPC); **Var**, Porquerolles, 13 m, N42.99534°, E6.2044°, ex *Bactrocera oleae* on *Olea europaea* (J.-C. Malausa & M. Thaon) (2 ♀) [NB377c/7090 (in GDPC), FAL1260/10438 (in FALPC)]; **Var**, Puget-Ville, 143 m, N43.26728°, E6.10671°, ex *Bactrocera oleae* on *Olea europaea* (J.-C. Malausa & M. Thaon) (1 ♂) [FAL1273a, not sequenced] (in GDPC). **ITALY: Liguria**, Bussana-Vecchia, N43.84026°, E7.82905°, 02.i.2012, emerged 24.ii.2012, ex *Myopites stylata* on *Dittrichia viscosa* (E. Spagnol) (2 ♀) [FAL1004/10130, FAL1075/10156] (in MNHN); (1 ♀) [GDEL4122/10194] (in FALPC) same data except collected 25.i.2011 and emerged iv.2011; (1 ♀) [FAL1415/10481] (in CBGP) same data except collected 06.vi.2012 and emerged 07.vi.2012; **Sardinia**, Province Oristano, N39.70041°, E8.739690°, 20.x.2012, sweeping on *Pistacia lentiscus* (L. Brancaccio & M. Thaon) (1 ♀) [FAL1508/10405] (in CNC); **Sardinia**, Province Oristano, N39.70041°, E8.739690°, 20.x.2012, emerged 24.x.2012, ex *Megastigmus pistaciae* on *Pistacia lentiscus* (L. Brancaccio & M. Thaon) (1 ♀) [FAL1513/10407] (in CNC).

### Eupelmus (Eupelmus) janstai

Taxon classificationAnimaliaHymenopteraEupelmidae

Delvare & Gibson

http://zoobank.org/27BFFF36-E3EE-4B94-AC0B-D4AB07836E1B

Eupelmus (Eupelmus) janstai Al khatib et al. (2014): 837–838.

#### Type material.

**Holotype** ♀. **CZECH REPUBLIC:** Břeclav district, Pavlov, 48.86750°N, 16.65416°E, sweeping on *Tilia platyphyllos*, 03.vii.2010 (G. Delvare) [GDEL4046/10032] (in MNHG). **Paratypes.** Moravia, Vranov riv., Dyje, 48.89472°N, 15.81250°E, 13.viii.1991, riparian forest (L. Masner) (2 ♀) (in CNC).

### Eupelmus (Eupelmus) longicalvus

Taxon classificationAnimaliaHymenopteraEupelmidae

Al khatib & Fusu

http://zoobank.org/F83B5381-0448-4EE3-B42F-F9AD7749F964

Eupelmus (Eupelmus) longicalvus Al khatib et al. (2014): 838–841.

#### Type material.

**Holotype** ♀. **SWEDEN:** Gotlands, Go, Gotlands kommun, Roleks, grazed calcareous pine forest. 57°32.207'N, 18°20.273'E, 16.vii–02.viii.2004, Trap ID 28, Coll. event 1458 (SMTP) [LF.ma.SW 02/10429] (in NHRS). **Paratypes. FRANCE: Alpes-Maritimes**, La Bollène-Vésubie, 1700 m, N43.96778°, E7.38111°, 19.vii.2009 (G. Delvare) (2 ♀) [GDEL4196/10606, GDEL4197/10607] (in GDPC); **Aveyron**, Peyreleau, 850 m, N44.17528°, E3.23750°, 22.vi.2009 (G. Delvare) (1 ♀) [GDEL4194/10604] (in GDPC); **Hautes-Alpes**, Mont-Dauphin, 1869 m, N44.68972°, E6.6786°, 18.viii.2008 (G. Delvare) (1 ♀) [GDEL4199/10609] (in FALPC). **ITALY: Friuli Venezia Giulia**, Giulia, Chiusaforte, 1450 m, N46.40527°, E13.4450°, 12.vii.2008 (G. Delvare) (1 ♀) [GDEL4038/10019] (in GDPC); **Friuli Venezia Giulia**, Giulia, Chiusaforte, 1380 m, N46.39944°, E13.45944°, 12.vii.2008 (G. Delvare) (1 ♀) [GDEL4191/10603] (in FALPC). **SWEDEN: Södermanland**, **Sö**, Södertälje kommun, Tullgarns näs, Rävsalaviken, mixed forest next to pasture, N58.955217°, E17.607550°, 03.vii/19.viii.2004, Trap ID 30, Coll. event ID 1055 (SMTP) (4 ♀ not sequenced & 1 ♀ sequenced) [LF.ma.SW 01/10428]; same data but 16.vi/17.vii.2005 and coll. event ID 1717 (5 ♀); same data but 17.vii/08.ix.2005 and coll. event ID 1718 (4 ♀ not sequenced & 1 ♀ sequenced) [LF.ma.SW 03/10430] (in NHRS and AICF); **Södermanland, Sö**, Huddinge kommun, Pine forest with garbage, N59.1765333°, E17.9938500°, 13.vii/10 viii 2004, Trap ID 5, Coll. event ID 766 (SMTP) (1 ♀not sequenced) (AICF).

### Eupelmus (Eupelmus) minozonus

Taxon classificationAnimaliaHymenopteraEupelmidae

Delvare

http://zoobank.org/B0EC22D2-129B-4A37-BF5C-FAE526CA3439

Eupelmus (Eupelmus) minozonus Al khatib et al. (2014): 843–846.

#### Type material.

**Holotype** ♀. **HUNGARY:** Veszprém, Hegyesd, 175 m a.s.l, 46.93333°N, 17.52278°E, 27.vi.2010, sweeping *Quercus cerris* (G. Delvare) [GDEL4030/10010] (in MNHG). **Paratypes.** Same data as holotype (4 ♀) [GDEL4030/10009, GDEL4030/10120 & GDEL4031/1001 (in GDPC and MNHN), GDEL4030/10668 (in FALPC)].

### Eupelmus (Eupelmus) opacus

Taxon classificationAnimaliaHymenopteraEupelmidae

Delvare

http://zoobank.org/23647401-C103-4AD7-ABA5-C41EC6126087

Eupelmus (Eupelmus) opacus Al khatib et al. (2014): 846–847.

#### Type material.

**Holotype** ♀. **SWEDEN:** Östergötland, Ög, Ödeshögs kommun, Omberg, Stocklycke äng, lime meadow, 58°18.452'N, 14°37.859'E, 23.viii/16.ix.2005, Trap ID 13, Coll. event 1648 (SMTP) [LF.ur.SW 02/10460] (in NHRS). **Paratype. GREECE:** Kerkini Lake N. Park, Kerkini, Krousia Mts site, Malaise tr., 06.vi–12.vii.2007, 41°11’32.4”N, 23°03’59.5”E, 190 m a.s.l., Leg. Gordon Ramel (1 ♀) [LF.ur.GR 01/10459] (in AICF).

### Eupelmus (Eupelmus) pistaciae

Taxon classificationAnimaliaHymenopteraEupelmidae

Al khatib

http://zoobank.org/111A405E-470F-481A-A43D-663460CEC078

Eupelmus (Eupelmus) pistaciae Al khatib et al. (2014): 847–850.

#### Type material.

**Holotype** ♀. **FRANCE:** Hérault, Cazevieille, 230 m a.s.l, 43.75222°N, 3.77000°E, 28.x.2011, emerged v.2012, ex *Megastigmus pistaciae* on *Pistacia terebinthus* (G. Delvare) [GDEL4027/10507] (in MNHG). **Paratypes. FRANCE: Hérault**, same data as holotype (6 ♀) [GDEL4027/6390 (in GDPC), GDEL4027/6391 (in FALPC), GDEL4027/6392 (in AICF), GDEL4027/6393 (in MNHN), GDEL4027/10004 (in FALPC), GDEL4027/10506 (in BMNH)] (3 ♂) [GDEL4027/6394 & GDEL4027/6395 (in GDPC), GDEL4027/10005 (in FALPC)]; **Hérault**, Viols-le-Fort, 200 m, N43.74583°, E3.70389°, 28.x.2009, emerged v. 2010, ex *Megastigmus pistaciae* on *Pistacia terebinthus* (G. Delvare) (1 ♀) [GDEL4022/3704bis] (in GDPC).

### Eupelmus (Eupelmus) priotoni

Taxon classificationAnimaliaHymenopteraEupelmidae

Delvare

http://zoobank.org/3565E30B-92D9-4DC4-BB94-7224D6BA3D22

Eupelmus (Eupelmus) priotoni Al khatib et al. (2014): 850–852.

#### Type material.

**Holotype** ♀. **FRANCE:** Aveyron, Sauclières, 700 m a.s.l, Lit de la Virenque, 43.96389°N, 3.35583°E, 15.vi.2011 (G. Delvare) [GDEL4051/10038] (in MNHG).

Unfortunately, a mistake was included in the original description of *Eupelmus priotoni* in Al khatib et al. (2014). Like other *Eupelmus* species described in this paper, the upper surface of the costal cell on the fore wing has only one row of setae on the apical half and not 3-4 rows as previously written.

### Eupelmus (Eupelmus) purpuricollis

Taxon classificationAnimaliaHymenopteraEupelmidae

Fusu & Al khatib

http://zoobank.org/1F354047-F041-4CD6-B1DA-1D53815B9B7C

Eupelmus (Eupelmus) purpuricollis Al khatib et al. (2014): 854–855.

#### Type material.

**Holotype** ♀. **GREECE:** Kerkini lake nr Neo Petritsi; Malaise trap, Midway Site, 30.vi–06.vii.2008, 41°18’49.8”N, 23°16’35.6”E, 750 m a.s.l., Leg. Gordon Ramel, [LF.ur.GR 02/10650] (in AICF). **Paratypes. GREECE:** same data as for holotype but 09–13.vii.2008 (1 ♀ not sequenced). Kerkini Lake N. Park, Kerkini, Krousia Mts site, Malaise tr., 11–17.vii.2007, 41°11’32.4”N, 23°03’59.5”E, 190 m a.s.l., Leg. Gordon Ramel, (1♀) [LF.ur.GR 05/10653] (in AICF); same data but 18–24.vii.2007 (1♀) [LF.ur.GR 03/10651] (in GDPC).

### Eupelmus (Eupelmus) simizonus

Taxon classificationAnimaliaHymenopteraEupelmidae

Al khatib

http://zoobank.org/CCD5B87E-9C81-456A-9DD8-F737AE6AC2F3

Eupelmus (Eupelmus) simizonus Al khatib et al. (2014): 855–856.

#### Type material.

**Holotype** ♀. **FRANCE:** Ardèche, Les Vans, 175 m a.s.l., Lit du Granzon, 44.38722°N, 4.15444°E, 15.vii.2012, sweeping on *Quercus pubescens*, (G. Delvare) [GDEL4142/10297] (in MNHG).

### Eupelmus (Eupelmus) tremulae

Taxon classificationAnimaliaHymenopteraEupelmidae

Delvare

http://zoobank.org/3001FBF7-A5AF-4D65-A274-6673F34264F1

Eupelmus (Eupelmus) tremulae Al khatib et al. (2014): 856–857.

#### Type material.

**Holotype** ♀. **CZECH REPUBLIC**, Jindóichùv Hradec, Veselí nad Lužnicí, 1 km E of Charles University field station (Ruda), 422 m a.s.l, 49.15296°N, 14.70646°E, ex *Harmandia* sp. (Cecidomyiidae) on *Populus tremula*, 05.vi.2007, adult emergence 13.vi.2007 (P. Jansta) [PJ07003_1_1/10570] (in MNHG). **Paratypes.** Same data (1 ♀) [PJ07003_1_1/10569] (in MNHN) (1 ♂) [PJ07003_1_1/10571] (in MNHN).

## Supplementary Material

XML Treatment for Eupelmus (Eupelmus) confusus

XML Treatment for Eupelmus (Eupelmus) gemellus

XML Treatment for Eupelmus (Eupelmus) janstai

XML Treatment for Eupelmus (Eupelmus) longicalvus

XML Treatment for Eupelmus (Eupelmus) minozonus

XML Treatment for Eupelmus (Eupelmus) opacus

XML Treatment for Eupelmus (Eupelmus) pistaciae

XML Treatment for Eupelmus (Eupelmus) priotoni

XML Treatment for Eupelmus (Eupelmus) purpuricollis

XML Treatment for Eupelmus (Eupelmus) simizonus

XML Treatment for Eupelmus (Eupelmus) tremulae

## References

[B1] Al khatib F Fusu L Cruaud A Gibson G Borowiec N Rasplus J-Y Ris N Delvare G (2014) An integrative approach to species discrimination in the *Eupelmus urozonus* complex (Hymenoptera, Eupelmidae), with the description of 11 new species from the Western Palaearctic. Systematic Entomology 39: 806–862. doi: 10.1111/syen.12089

[B2] ICZN (2012) International Code of Zoological Nomenclature. Fourth Edition http://www.iczn.org/iczn/index.jsp [accessed 19 October 2014]

